# Supplementary material for: Effect of cosmetic hair treatment and natural hair colour on hair testosterone concentrations
Source: PLoS One. 2023 Oct 11;18(10):e0291812. doi: 10.1371/journal.pone.0291812 (PMC10566713; doi:10.1371/journal.pone.0291812)
Supplement: Attachment — Submitted filename: Response to reviewers.docx [file pone.0291812.s003.docx]

Response to reviewers

**Reviewer #1**: The study entitled “Effect of cosmetic hair treatment and natural hair colour on hair testosterone concentrations” submitted for publication in Plos One took the hair samples from the initial clinical study and reevaluated the hair testosterone concentrations solely. Thereby hair testosterone concentrations were compared between two different sample collection time points (3 months apart) and analyzed for potential differences resulting from natural hair color or cosmetic hair treatment. The results are described sufficiently, and the statistical tests were appropriate. However, overall, the study can only be considered preliminary as only observations were provided that do not allow (yet) for a final conclusion. I miss some experiments that would allow for detailed conclusions, e.g., applying an additional analytical method (e.g., mass spectrometry) that would allow differentiating between actual in vivo effects or artificial analytical increases of the testosterone concentrations. Also, additional in vitro experiments would have allowed the differentiation of different hair treatment procedures against untreated hair. At least regarding the use of mass spectrometry methods, the authors discuss in detail why such analysis where not feasible. But without further experiments, the data and results seem too small to be published in a full-length original research article. Publication of the presented observations might be better suited as a short communication or technical note maybe in a different journal.

**Reply: We appreciate that Reviewer #1 correctly describes the context of our observational study with repeated hair samples from 146 women. A future study with an experimental study design is needed to assess and provide more data on the effects of in vivo and peri-analytical factors on hair testosterone concentrations in women.**

Some minor comments:

Introduction: Please go into more detail about why testosterone should be measured in hair.

Fig 2 and 3 could be combined in just one figure.

**Reply: The first paragraph of the introduction has been altered according to the comment. Figure 3 consists of a paired subset of the participants (n=24), while Figure 2 consists of the whole cohort (n=191), which is the reason why the figures cannot be combined.**

Reviewer #2: The manuscript is well written and the analysis adequate. A drawback might be the used methodology (immunoassay) compared to studies that have used LC-MS/MS. But this fact is extensively discussed in the discussion. Therefore I recommend the manuscript for publication.

**Reply: Thank You for appreciating our work.**
